# An investigation to validate the equivalence of physes obtained from different anatomic regions in a single animal species: Implications for choosing experimental controls in clinical studies

**DOI:** 10.1016/j.bonr.2019.100209

**Published:** 2019-05-05

**Authors:** Steven Widmer, Richard P. Steiner, Melanie A. Morscher, Mark Shasti, Dennis S. Weiner, Mark J. Adamczyk, Robin DiFeo Childs, William J. Landis

**Affiliations:** a Wooster Orthopaedics and Sports Medicine Center, Wooster, OH, USA; b The University of Akron, Akron, OH, USA; c Akron Children's Hospital, Akron, OH, USA; d Norton Leatherman Spine Center, Louisville, KY, USA; e University of California, San Francisco, San Francisco, CA, USA

**Keywords:** Physeal equivalence, Growth plate, Gene expression, Aggrecan, Type II collagen

## Abstract

Control tissue in studies of various orthopedic pathologies is difficult to obtain and presumably equivalent biopsies from other anatomic sites have been utilized in its place. However, for growth plates, different anatomic regions are subject to dissimilar mechanical forces and produce disproportionate longitudinal growth. The purpose of this study was to compare gene expression and structure in normal physes from different anatomic regions within a single animal species to determine whether such physes were equivalent. Thirteen female New Zealand white rabbits (five 15-week-old and eight 19-week-old animals) were euthanized and physes harvested from their proximal and distal femurs and proximal tibiae. Harvested physes were divided into groups for histological, immunohistochemical (IHC), and reverse transcription-quantitative polymerase chain reaction analyses. All physes analyzed demonstrated no apparent differences in morphology or proteoglycan staining intensity on histological examination or in type II collagen presence determined by IHC study. Histomorphometric measures of physeal height as well as gene expression of type II collagen and aggrecan were found to be statistically significantly equivalent (*p* < 0.05) among the three different bones from the total number of rabbits. Summary data suggest that the structural similarities and statistical equivalence determined among the various physes investigated in the rabbit validate these tissues in this species for use as surrogate controls by which physeal abnormalities may be compared and characterized in the absence of otherwise normal control tissues. Other species may exhibit the same similarities and equivalence among different physes so that such tissues may serve in like manner as controls for assessing a variety of orthopedic conditions, including those occurring in humans.

## 1 Introduction

The study of various orthopedic pathologies often compares abnormal and control tissue to characterize the type and extent of changes occurring in the former and to determine an appropriate treatment modality. In many cases, normal tissue or an equivalent tissue to serve as an experimental control is extremely difficult to obtain, if available at all, and especially so in situations concerning physeal tissue. Indeed with regard to physeal samples, it is known that growth plates from different anatomic regions are subject to dissimilar mechanical forces (Fishkin et al., 2006; Williams et al., 1999) and produce disproportionate longitudinal growth (Lovell and Winter, 1986; Maes and Kronenberg, 2012). Thus, such growth plates may not be equivalent in their structure, biochemistry, function or other characteristics and would also fail as a proper control comparing another growth plate from a different anatomic region in the same animal or species. In a recent report by Lui et al. (Lui et al., 2018), the growth plates of smaller bones (metacarpals, phalanges) were quite different from the growth plates of larger bones (femurs, tibiae), especially in the aging process which contributes to their length inequalities.

On the other hand, in previous work examining the proximal femoral physis, for example in slipped capital femoral epiphysis (SCFE), biopsy samples obtained from other anatomic sites (the distal femoral and proximal tibial and fibular physes) in a different orthopedic condition were utilized as suitable control tissues (Scharschmidt et al., 2009). This study demonstrated that, despite the various forces and specific functional nature of a growth plate from different anatomic regions, seemingly disparate growth plates from long bones were in fact equivalent in certain aspects of structure, biochemistry and function and acceptable as control specimens for SCFE comparisons. In this context of control tissues, it is imperative as part of the experimental design of an investigation that physes from separate anatomic regions be as similar as possible to the tissue of interest so as to serve as appropriate control material. In the present work, an approach is documented by which comparisons can be made in structure and gene expression in normal physes from different anatomic regions in a single species to determine whether such physes are equivalent and may be utilized as control tissues in investigations relevant to physeal abnormalities in orthopedics.

The work that follows rests on structural, genetic and animal model analyses to validate physes as equivalent in various anatomically distinct sites from rabbits. Such animals are often utilized as surrogates to humans since structural and molecular (genetic) investigations of normal and diseased human growth plate tissue are very limited, as noted above, although they are becoming more prevalent in literature reports. Ballock and O'Keefe (Ballock and O'Keefe, 2003), for instance, established that chondrocyte hypertrophy is important in longitudinal growth, matrix synthesis, and chondrocyte proliferation, and Scharschmidt et al. (Scharschmidt et al., 2009) demonstrated that type II collagen and aggrecan levels are decreased in patients with SCFE. Others have shown chondrocyte disorganization, diminished overall cell counts, and increased apoptosis in SCFE growth plates (Agamanolis et al., 1985a; Agamanolis et al., 1985b; Ippolito et al., 1981; Adamczyk et al., 2005).

Related to these examples of structural and genetic studies of physes, various animal models have been coincidentally established to study growth plates in their healthy and diseased states. In this regard, different animal models have shown similarity to human models in demonstrating altered microscopic architecture under mechanical compression (Hosseinzadeh and Milbrandt, 2016; Alberty et al., 1993; Stokes et al., 2007; Rudicel et al., 1985; Amini et al., 2010). More recently, Bries et al. (Bries et al., 2012) reported that, as in human physes (Scharschmidt et al., 2009), there was decreased gene expression of aggrecan, type II and type X collagen in rabbit physes subjected to compression. Miniature swine are also an attractive animal model in that swine femoral anatomy is similar to that of humans (Salter, 1980; Douglas and Rang, 1981), and analysis of proximal femurs by Tank et al. (Tank et al., 2013) demonstrated gene expression differences in hypothyroid and control miniature swine with results yielding insight into the hypothyroid condition in humans.

Some studies cited above could not have been conducted without a comparison between experimental tissues and appropriate control specimens carefully validated as relevant to the experimental unknowns. The present study, then, has undertaken a means to verify the equivalence of physes obtained from different anatomic regions in a single animal species, the rabbit, so that the tissues may be taken as controls to compare and characterize orthopedic physeal abnormalities. Histological and gene expression analyses have been applied to test and potentially validate the hypothesis that normal physes from different anatomic locations in an individual species (the rabbit) have no apparent morphological differences on histological examination and are statistically equivalent in physeal height and the expression levels of two prominent extracellular matrix genes (type II collagen and aggrecan).

## 2 Methods

### 2.1 Samples and sample preparation

Normal animals used in this investigation were approved for studies by the Institutional Animal Care and Use Committee at the Northeast Ohio Medical University (Rootstown, OH) and according to the policies outlined by the National Institutes of Health Guide for the Care and Use of Laboratory Animals (NIH Publications No. 8023, revised 1978). In the present work, thirteen normal female New Zealand white rabbits (five 15-week-old animals; eight 19-week-old animals) (Bries et al., 2012) were euthanized and growth plates harvested from their proximal femoral heads and distal femurs and proximal tibiae. The harvested physes were divided into portions for histological, immunohistochemical (IHC), and reverse transcription-quantitative polymerase chain reaction (RT-qPCR) analyses. Specimens for histological and IHC studies were placed in vials containing 10% neutral buffered formalin (NBF; VWR Intl., Radnor, PA) and those for RT-qPCR analysis were immersed in sterile tubes containing RNA*later*™ (Ambion, Thermo Fisher Scientific, Inc., Waltham, MA) and stored in a −80 °C freezer.

Rabbit bone samples stored in NBF were washed in phosphate buffered saline, decalcified in formic acid, embedded in paraffin and sectioned at 6 μm thickness using a Leica model RM 2255 microtome (Leica Microsystems, Richmond, IL). Subsequently, mounted sections were deparaffinized and rehydrated for either histological staining or IHC analysis (Bries et al., 2012; Tank et al., 2013).

### 2.2 Histology and histomorphometry

For histological analysis, rabbit sections were stained with Periodic Acid Schiff (PAS) for the presence of proteoglycans. Histomorphometric measurements (*n* = 5 sections from each bone) from three matched sets (proximal femoral, distal femoral, proximal tibial) of rabbit growth plate height/thickness were obtained from PAS-stained sections across the full transverse growth plate width of the tissue as described in a previous study (Tank et al., 2013).

### 2.3 Immunohistochemistry

Chondrocyte extracellular matrix formation was assessed by means of IHC analysis of type II collagen in rabbit bone sections (II-II6B3; Developmental Studies Hybridoma Bank at the University of Iowa, Iowa City, Iowa) on a Leica Bond Max (Leica Microsystems) with use of an open polymer detection system (Leica Microsystems) (Bries et al., 2012). Briefly, prepared sections were treated with 0.1% pronase for 20 min at 37 °C, blocked with Background Buster (INNOVEX, Richmond, CA) for 1 h and subsequently exposed and bound for 1 h to the primary antibody to type II collagen (3 μg/ml). Detection of type II collagen was achieved by means of a polymer link reaction with 3,3′-diaminobenzidine (MaxTag DAB tablets, Rockland, Limerick, PA) for 5 min followed by hematoxylin counterstaining (Bries et al., 2012). Appropriate negative control slides without primary antibody accompanied positive slides during each Bond Max program. Stained histological or immunohistochemical sections were examined and photographed with use of an Olympus IX-70 light microscope and MicroSuite software (Olympus America, Melville, NY) (Bries et al., 2012; Tank et al., 2013).

### 2.4 Gene expression analyses

**Fig. 1:**
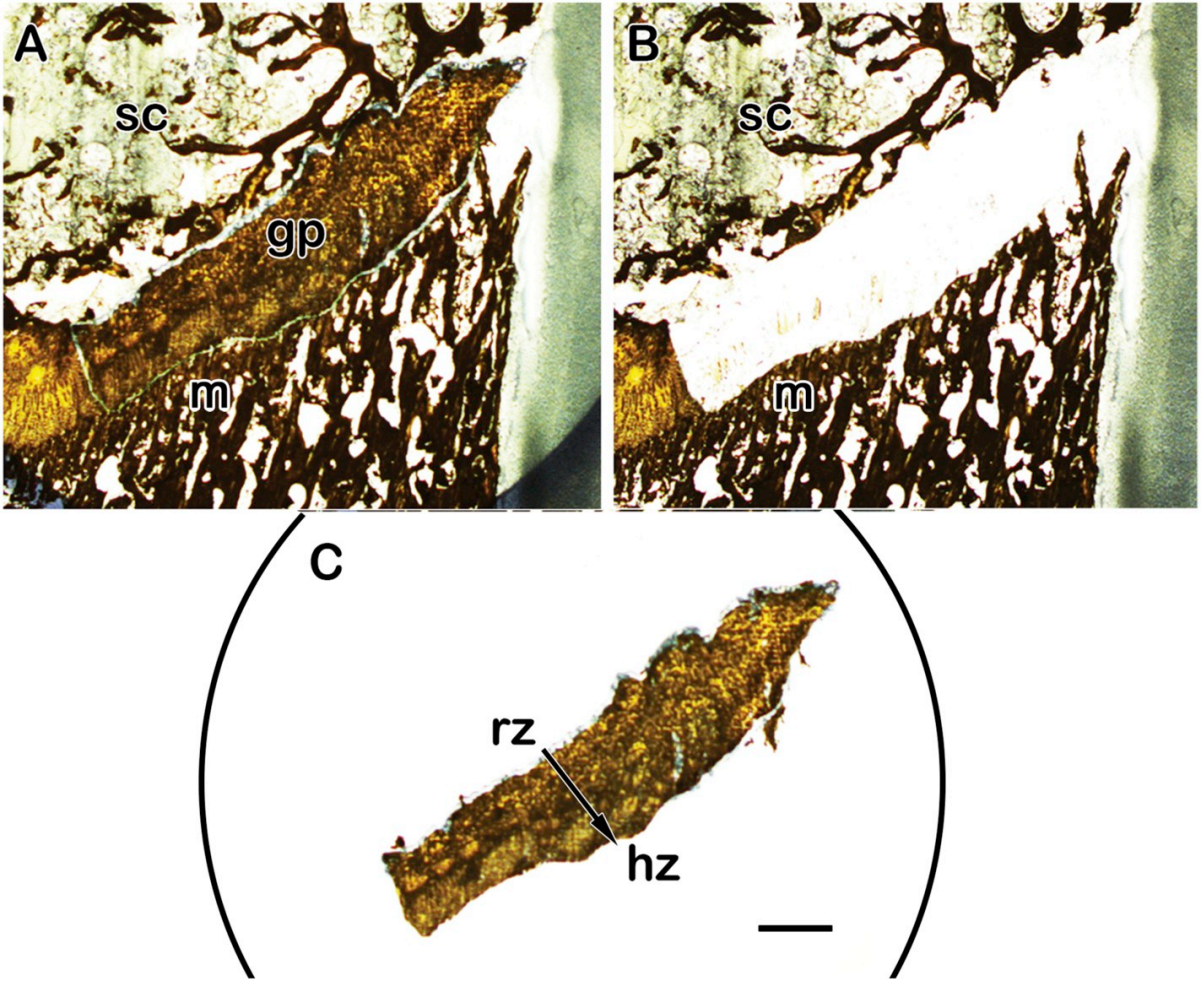
A representative example of a growth plate isolated by LCM. Frozen sections were prepared (10–12 μm thick), briefly fixed in 70% ethanol, stained with eosin, and dehydrated with xylene. (A) A lightly stained intact cryosection observed under appropriate magnification in the LCM system equipped with an automated stage; the subchondral bone (sc), growth plate (gp) and mineralized metaphysis (m) were identified and the growth plate was outlined. (B) Image of (A) with growth plate removed by use of laser cutting along its outlined perimeter; the process anneals the growth plate to a polymer substrate applied to an LCM cap, which is then lifted free of the cryosection. (C) Image of the captured growth plate on the LCM cap and showing the entirety of the chondrocyte resting zone (rz) to hypertrophic zone (hz) of the growth plate. Scale bar = 0.5 cm for all images.

Levels of gene expression of aggrecan, type II collagen, SOX9 (sex-determining region Y – box 9), ß-actin and 18S rRNA were determined by RT-qPCR for all samples stored in RNA*later*™. Gene primer sets were designed, tested and previously published for each species except SOX9 for rabbit (Table 1) (Bries et al., 2012). Frozen rabbit bones consisting of proximal femurs, distal femurs, and proximal tibiae (*n* = 13 each) were ground to powders separately under liquid nitrogen in a Spex 6750 freezer/grinder mill (Spex SamplePrep, Metuchen, NJ). Total RNA was subsequently isolated, purified, quantified and reverse-transcribed following published methods (Bries et al., 2012). Growth plate chondrocytes were obtained by laser capture microdissection (LCM) from one rabbit sample set of proximal femur, distal femur and proximal tibia and expression of the genes of interest above from these cells was independently assayed. An example of a single animal growth plate isolated by LCM is depicted in Fig. 1. Briefly for LCM procedures, each rabbit physis was frozen-sectioned at 10-12 μm thicknesses onto PEN membrane slides (Thermo Fisher Scientific, Inc.) following a decalcification in an RNA solution (U.S. Patent No. 9,783,840). Tissue sections were then fixed in 70% ethanol for 1 min. Tissue section features were visualized with an eosin stain and intact regions of growth plates removed using infrared (IR) and ultraviolet (UV) lasers of an ArcturusXT™ LCM system (Arcturus, Thermo Fisher Scientific, Inc.). Total RNA was isolated and purified from captured chondrocytes utilizing the PicoPure kit (Arcturus, Thermo Fisher Scientific, Inc.) and reverse-transcribed as previously detailed (Scharschmidt et al., 2009; Tank et al., 2013).

**Table 1:** Rabbit-specific designed primer sequences.

Oligo name	Accession number (Genbank)	Sequence 5′ to 3′	Melting temperature (°C)	Amplicon length (base pairs)
SOX9 (sex determining region Y) – box 9	AY598935	F376 AGTACCCGCACCTGCACAAC R454 CGCTTCTCGCTCTCGTTCAG	85	79

Gene expression analysis of all samples was accomplished with an Applied Biosystems 7500 system (Applied Biosystems, Thermo Fisher Scientific, Inc.). All reverse-transcribed RNA retrieved from ground rabbit samples was processed utilizing the standard instrument protocol but the limited quantity of RNA from LCM samples had an extended step of 30 sec at 72 °C added to the standard protocol. Generated threshold values were converted to quantities by the relative standard curve methodology. 18S rRNA numbers were used to determine the quality of each sample and typically low quality or quantity samples were removed from the study. All ground samples in this study were of appropriate quality. The use of reference or housekeeping genes in the normalization calculations for the ground samples was verified by utilizing 18S rRNA in the LCM-isolated samples (Table 2). Chondrocytes were specifically isolated and fold changes that were approximately two or less were considered equivalent with consideration for a sample size of one. Gene expression values normalized to ß-actin and SOX9, used as housekeeping genes, were averaged and compared, and standard errors of resulting mean values were calculated for each of the respective sample types.

**Table 2:** RT-qPCR of chondrocytes isolated by LCM from growth plates (three locations) of a single rabbit to verify genes of interest.

Location	Normalized to 18S rRNA Fold change compared to proximal femur				Normalized to β-actin Fold change compared to proximal femur		
	Aggrecan	Type II collagen	β-actin	SOX9	Aggrecan	Type II collagen	SOX9
Proximal femur	1.0	1.0	1.0	1.0	1.0	1.0	1.0
Distal femur	0.60	0.57	0.89	0.57	1.05	1.13	0.83
Proximal tibia	1.67	1.40	0.80	1.05	1.53	1.16	0.99

LCM = laser capture microdissection; SOX9 = (sex determining region Y) – box 9.

### 2.5 Statistical analyses

Fold changes in expression levels of the two cartilage genes, type II collagen and aggrecan, were analyzed for statistical equivalence among growth plates of three regions of the primary bones of rabbit hind limbs: the proximal femur (PF), distal femur (DF), and proximal tibia (PT). Analyses were conducted for data normalized to SOX9. Fold changes in gene expression among the three growth plate regions were determined for each rabbit by dividing the larger (normalized) data value by the smaller (normalized) data value, that is,

$$\text{Gene expression ratio}.\frac{\text{Larger normalized data value}}{\text{Smaller normalized data value}}.$$

Gene expression ratios constructed in this way will always be ≥ 1, regardless of which of the growth plate regions being compared produces the larger data value. For example, suppose a rabbit had a normalized value of 1.27 for the PF and 1.32 for the DF. Its gene expression ratio from above would be 1.32/1.27 = 1.04. Then consider a rabbit with normalized values of 1.79 and 0.89 for PF and DF, respectively. Here its gene expression ratio would be 1.79/0.89 = 2.01. In the first case, the DF had the larger data value, while PF was larger in the second case. In both cases, the gene expression ratios were ≥ 1, so that one can think of the gene expression ratio as an absolute fold change between the locations of the rabbit primary bones.

For this study, in eukaryotic gene expression, a mean fold change of 2 or larger (≥ 2) was considered the minimum for physiological significance following guidelines established in the Real Time PCR Handbook and Precision in the qPCR technical note from Applied Biosystems (www.thermofisher.com). Therefore, a mean fold change of < 2 was considered biologically equivalent. Gene expression from two growth plate regions was tested for equivalence using a *t*-test of the statistical hypotheses:

$$H_{0}:\mu =2\;\mathrm{vs}.\;H_{1}:\mu \;<\;2,$$

where μ is the population mean gene expression ratio for the two locations (H) being compared. Rejection of the null hypothesis indicates equivalence in gene expression between the two growth plate regions. Three gene expression ratios are needed to compare the three growth plate regions: PF vs. DF, PF vs. PT, and DF vs. PT. Each gene expression ratio was tested as described above, producing a family of three conclusions, one for each test. The familywise level of significance used was 0.05. Thus, the probability of falsely claiming equivalence on at least one of the tests in the family was limited to 0.05. To achieve such probability, a Bonferroni correction was applied, resulting in a level of significance of 0.0167 for each comparison in the family. In addition to testing for equivalence, an upper bound for each of the three gene expression ratios was computed with a 95% confidence level.

Following an analogous approach to the analysis of gene expression, statistical equivalence of growth plate height was also determined except that here a mean fold change of 1.8 or larger was considered biologically meaningful. Therefore, a mean fold change of < 1.8 was considered biologically equivalent (Tank et al., 2013). The height of two growth plate regions was tested for equivalence using a *t*-test of the statistical hypotheses:

$$H_{0}:\mu =1.8\;\mathrm{vs}.\;H_{1}:\mu \;<\;1.8,$$

where μ is the population mean growth plate height ratio for the two locations (H) being compared.

Finally, a measure of agreement (R.P. Steiner, personal communication) was computed for each pair of growth plate regions. This measure is based on the sum of squared differences (*d*) in normalized values between two regions being compared. The general form of the measure is:

$$1-\frac{\text{Observed}\;\text{disagreement}}{\text{Random}\;\text{or}\;\text{chance}\;\text{disagreement}}.$$

The particular measure used in this study is called A_3_. It is calculated as

$$A_{3}=1-\frac{\sum_{i=1}^{n}d_{i}^{2}/n}{\sum_{i=1}^{n^{2}}d_{i}^{2}/n^{2}}$$

The observed disagreement in the fraction above is the sum of the actual squared differences between locations for all rabbits. For the measure of random or chance disagreement, each normalized value from one location is paired with each normalized value from the other location. The difference (*d*) for each of these is *n*^*2*^ pairs. A value of 0 for A_3_ indicates agreement no better than chance, while a value of 1 indicates perfect agreement. Negative values indicate poorer agreement than might be expected by chance.

Given the two different aged groups of rabbits and the individual animals comprising each group, age and bone growth variations were tested for equivalence in each group of animals (five 15-week-old rabbits; eight 19-week-old rabbits). Data for each group were then compared. No statistically significant differences in equivalence were found (data not shown) and the specimens were subsequently pooled (*n* = 13) for further statistical analyses of type II collagen and aggrecan gene expression levels.

## 3 Results

### 3.1 Histology and histomorphometry

**Fig. 2:**
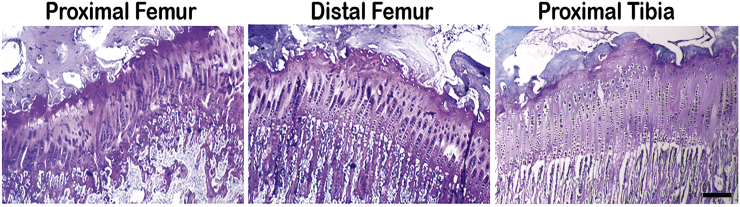
Light micrographs of representative histological sections of physes obtained from the hind limbs of normal, 15-week-old rabbits and stained with Periodic Acid Schiff (PAS) for the presence of proteoglycans. Tissue sections were taken from three different anatomic regions: proximal femur, distal femur, and proximal tibia. Rabbit growth plate chondrocytes were normal in size and shape within their lacunae, organized developmentally and progressively in a proximal-to-distal direction from resting to proliferative to hypertrophic cells. PAS staining was uniform in intensity and distribution in all the physes. Scale bar = 200 μm for all panels.

All rabbit physes analyzed (proximal and distal femur and proximal tibia) demonstrated no apparent morphological differences on histological examination. PAS staining intensity for proteoglycans was qualitatively similar and all physes were consistently characterized by orderly linear and generally parallel stacks of chondrocytes (chondrons), cell structure and lacunae without unusual irregularities in size and shape, and uniform staining of extracellular matrix surrounding chondrocytes (Fig. 2). In addition, chondrocytes were found in physes in normal proximal-to-distal development and progression from smaller and more immature resting cells to proliferative cells in chondrons to larger hypertrophic cells bordering the mineralization front at the tissue metaphyses (Fig. 2).

**Fig. 3:**
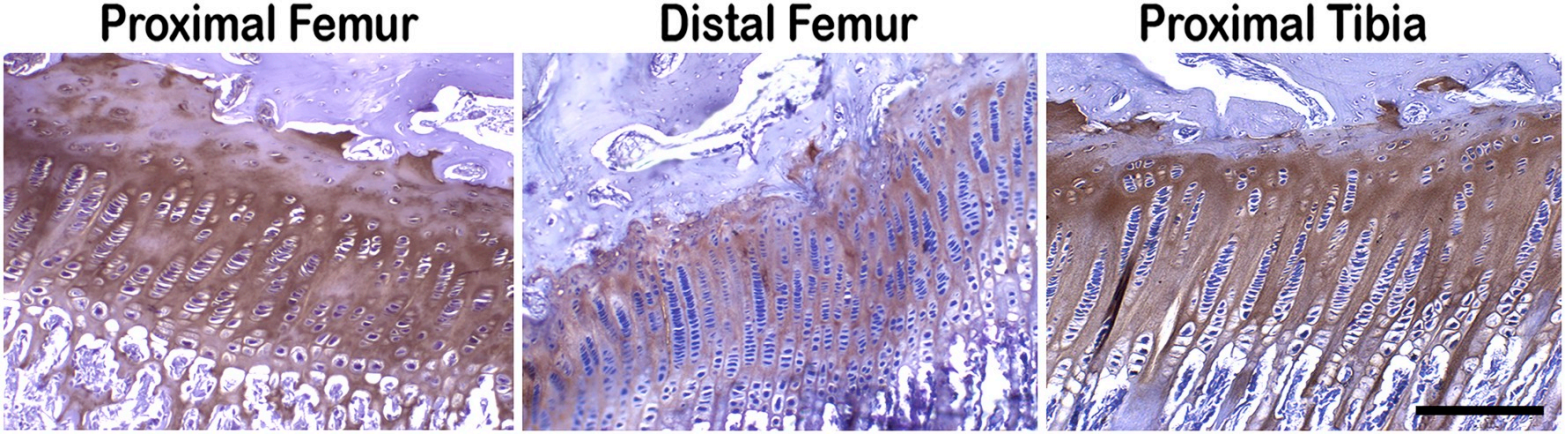
Light micrographs of representative immunohistological sections of physes obtained from the hind limbs of normal, 15-week-old rabbits, stained tan/brown (DAB) by IHC for the presence of type II collagen and counterstained with hematoxylin. Sections were taken from proximal femur, distal femur, and proximal tibia. As with PAS staining histologically (Fig. 2), immunostaining for type II collagen was found to be similar in its pattern and intensity of staining between growth plates. There was a qualitative increase in type II collagen staining in each physeal specimen in the upper proliferative regions of the growth plates. In addition, regions of hypertrophy in each specimen appeared with a qualitative decrease in type II collagen staining. Scale bar = 200 μm for all panels. (For interpretation of the references to colour in this figure legend, the reader is referred to the web version of this article.)

Type II collagen IHC examination of the rabbit specimens showed no remarkable differences in the three physes (proximal and distal femur and proximal tibia; Fig. 3). Type II collagen was generally similar in distribution across all growth plates and followed its known pattern during endochondral ossification. In this regard, there appeared to be a qualitative increase in type II collagen staining in the upper proliferative zones of cells at each anatomic site in the rabbits. Hypertrophic cell regions in these animals were marked qualitatively by decreased type II collagen staining (Fig. 3).

### 3.2 Statistical analyses of gene expression and growth plate height

**Fig. 4:**
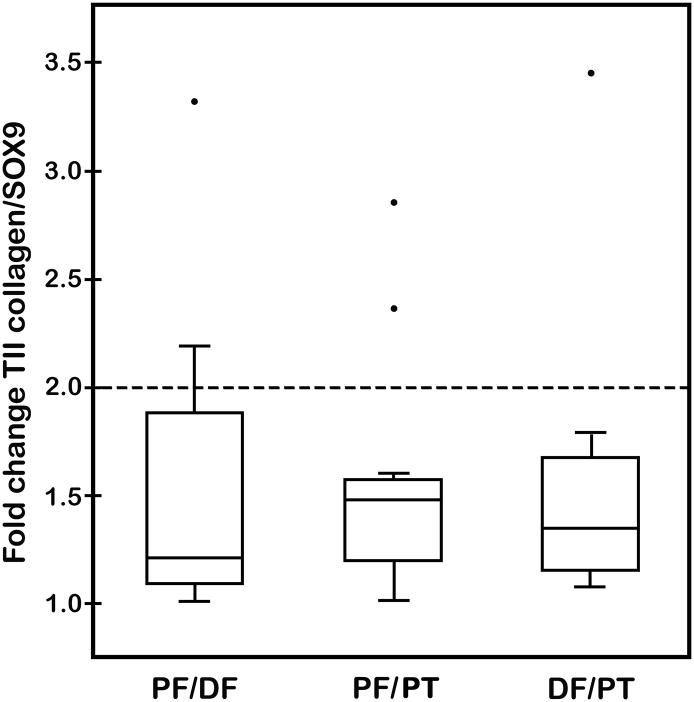
Box-and-whiskers plot of fold change in gene expression levels for type II collagen normalized to SOX9 for rabbits (*n* = 13). Statistical comparisons were made between physes from the PF and DF, PF and PT, and DF and PT. Fold change value of 2 is marked as a dashed line in the plot and each comparison is shown by a box with upper and lower bounds about a mean fold change value. The median fold change value is represented by the single line within the box. Errors are expressed as standard errors of mean fold change values. Four outlier points were measured with fold change > 2. Statistical analyses to determine possible equivalence of data are presented in Table 3.

**Fig. 5:**
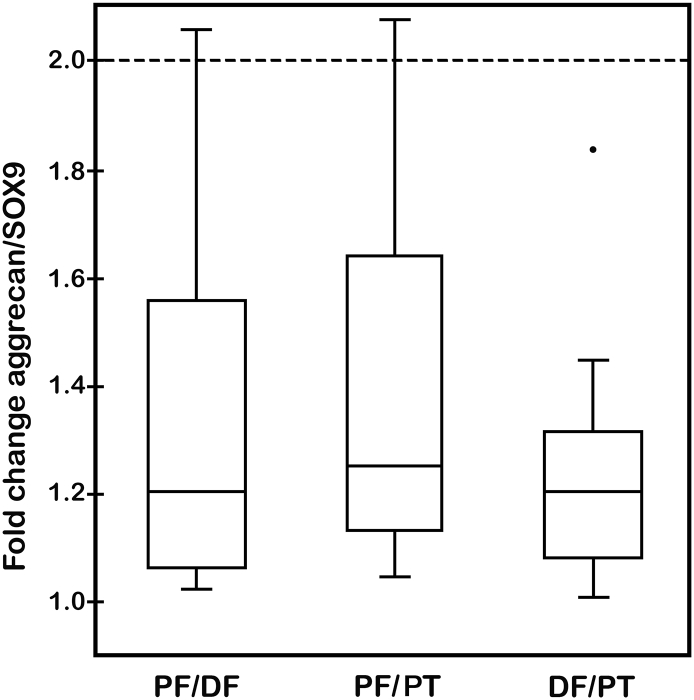
Box-and-whiskers plot of fold change in gene expression levels for aggrecan normalized to SOX9 for rabbits (*n* = 13). Statistical comparisons were made between physes from the PF and DF, PF and PT, and DF and PT. Fold change value of 2 is marked as a dashed line in the plot and each comparison is shown by a box with upper and lower bounds about a mean fold change value. The median fold change value is represented by the single line within the box. Errors are expressed as standard errors of mean fold change values. There was a single outlier point measured with fold change of ~1.85 for DF vs. PT. Statistical analyses to determine possible equivalence of data are presented in Table 4.

Results of statistical analyses of the fold change in gene expression levels of type II collagen and aggrecan are presented in Table 3/Fig. 4 and Table 4/Fig. 5, respectively, for all rabbits (*n* = 13) from the two aged groups (15- and 19-weeks-old). When normalized with SOX9, the fold changes in expression levels of both type II collagen (Fig. 4) and aggrecan (Fig. 5) were shown in box-and-whisker plots to be statistically equivalent, that is, the mean fold changes in gene expression were < 2 (*p* < 0.05). The upper bounds for the mean fold changes ranged from 1.86 for type II collagen (PF vs. DF) (Fig. 4) to 1.35 for aggrecan (DF vs. PT) (Fig. 5). Agreement based on the coefficient A_3_ ranged from 31% to 54% better than chance for type II collagen (Table 3) and from 25% to 37% better than chance for aggrecan (Table 4). Tests for equivalence for mean fold change in gene expression levels were conducted for both type II collagen and aggrecan and demonstrated equivalence among the growth plates compared (Table 3, Table 4, respectively).

**Table 3:** Tests of equivalence for mean fold change in gene expression levels of type II collagen (normalized to SOX9) between rabbit growth plates of proximal femur (PF), distal femur (DF), and proximal tibia (PT).

Comparison	Mean fold change	SEM	*p*-value for test of fold change < 2	Upper bound on mean fold change (95% confidence)	Agreement based on mean squared difference (A_3_)
PF vs. DF	1.53	0.183	0.0128*	1.86	0.538
PF vs. PT	1.54	0.145	0.0040*	1.80	0.440
DF vs. PT	1.52	0.177	0.0096*	1.84	0.314

(*) indicates statistical equivalence for growth plates compared (*n* = 13).

**Table 4:** Tests of equivalence for mean fold change in gene expression levels of aggrecan (normalized to SOX9) between rabbit growth plates for proximal femur (PF), distal femur (DF), and proximal tibia (PT).

Comparison	Mean fold change	SEM	*p*-value for test of fold change < 2	Upper bound on mean fold change (95% confidence)	Agreement based on mean squared difference (A_3_)
PF vs. DF	1.32	0.089	< 0.0001*	1.48	0.374
PF vs. PT	1.39	0.091	< 0.0001*	1.55	0.281
DF vs. PT	1.24	0.060	< 0.0001*	1.35	0.247

(*) indicates statistical equivalence for growth plates compared (*n* = 13).

**Fig. 6:**
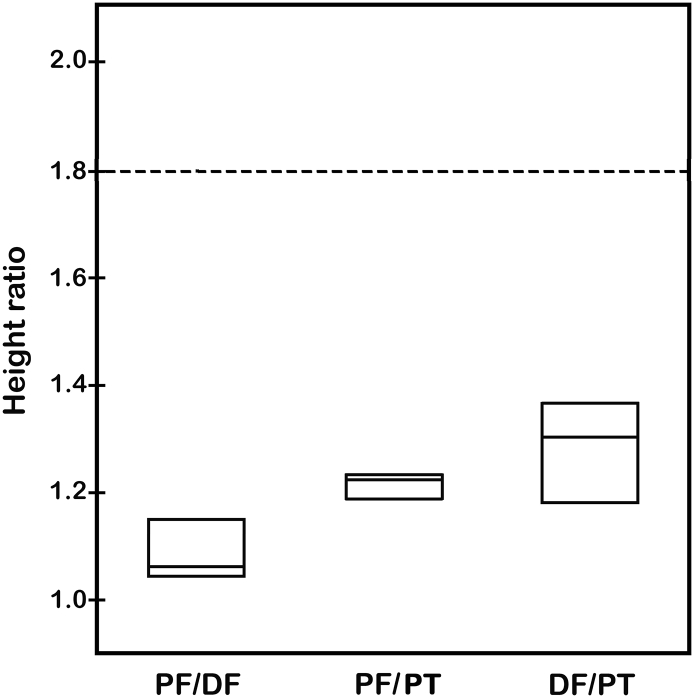
Box-and-whiskers plot of height ratios of physes for rabbits (*n* = 3). Statistical comparisons were made between physes from the PF and DF, PF and PT, and DF and PT. Fold change value of 1.8 is marked as a dashed line in the plot and each comparison is shown by a box with upper and lower bounds about a mean fold change value. The median fold change value is represented by the single line within the box. Errors are expressed as standard errors of mean fold change values and in these examples are too small to be apparent. Statistical analyses to determine possible equivalence of data are presented in Table 5.

As with the fold changes in expression levels for type II collagen and aggrecan, growth plate heights of the various bone samples were also shown to be statistically equivalent. Box-and-whisker plots for the growth plate mean height ratios for all specimens were < 1.8 (*p* < 0.05) (Fig. 6). The upper bounds for the mean height ratios ranged from 1.18 (PF vs. DF) to 1.34 (DF vs. PT) (Fig. 6). Agreement based on the coefficient A_3_ ranged from 45% worse than chance (PF vs. PT) to 36% better than chance (PF vs. DF) (Table 5). There were only *n* = 3 rabbits for this analysis, and the agreement measure was especially sensitive to outlier data points of the small sample size in this instance. This situation was reflected in the resulting negative values of A_3_ on comparing PF vs. PT and DF vs. PT (Table 5). Equivalence was demonstrated in rabbit growth plate mean height ratios among the samples compared (Table 5).

**Table 5:** Tests of equivalence between rabbit growth plate mean height ratios for proximal femur (PF), distal femur (DF), and proximal tibia (PT).

Comparison	Mean height ratio	SEM	*p*-value for test of height ratio < 1.8	Upper bound on mean height ratio (95% confidence)	Agreement based on mean squared difference (A_3_)
PF vs. DF	1.08	0.0325	0.0010*	1.18	0.363
PF vs. PT	1.21	0.0140	0.0003*	1.26	−0.446
DF vs. PT	1.28	0.0538	0.0053*	1.44	−0.435

(*) indicates statistical equivalence for growth plates compared (*n* = 3).

## 4 Discussion

The present study addresses an often critical issue in the investigation of clinical pathological specimens for which normal human tissue to be used as comparative control material is unavailable. In such cases, the clinical samples may be analyzed with respect to each other, but the approach is otherwise limited without reference to an acceptable normal standard. Circumventing this situation with human tissue that is equivalent to normal is an avenue that may be useful, but even this specimen type may be difficult to obtain and, furthermore, to validate. In investigations of orthopedic disorders or pathologies, particularly physeal abnormalities as in SCFE, hypothyroidism, dysplasias and a host of other atypical conditions, few studies, in the absence of normal samples, have examined material equivalent to normal.

In the work here, the question occurred as to whether the physes from different anatomic locations in a single species, despite the tissue experience with different mechanical forces and growth response (Fishkin et al., 2006; Williams et al., 1999; Lovell and Winter, 1986; Maes and Kronenberg, 2012; Lui et al., 2018) as well as specific function, could in fact be equivalent in structure, biochemical markers and other factors. If so, such physes could serve appropriately as control specimens in clinical studies comparing an abnormal physis, which could be biopsied, to a normal physis, which could not be interrogated. This study is the first to examine physeal structure and type II collagen and aggrecan gene expression for equivalence from three different anatomic regions in a single animal species, in this instance the rabbit. The experimental design of the current report provides an approach by which different physes may be carefully probed and documented in terms of their component structure and expression levels of principal genes, analyzed by stringent statistical means. Typical statistical analyses have examined differences through a *t*-test or ANOVA and assumed that without statistically significant changes the tissues were equivalent. This study has attempted to establish statistical equivalency based on major molecules of long bone growth plates and previously known statistical differences from their analyzed disease states (Scharschmidt et al., 2009; Bries et al., 2012; Tank et al., 2013). These data from the physes of the proximal and distal femurs and proximal tibiae of normal rabbits validate these seemingly distinct tissues as equivalent. Such physes, then, may be utilized with confidence as control tissues in study protocols that are directed toward characterizing the nature of abnormal physes on comparison with them.

In consideration of investigating samples of two different ages (15 and 19 weeks) and differences in the longitudinal bone growth of each individual rabbit, age and growth variations clearly occur for every animal. Thus, equivalence of data such as gene expression by such samples would be expected to vary with time as well. In this case, statistical equivalence was calculated for each of the two aged groups of rabbits and then compared between the two groups. Since no statistically significant differences in equivalence were found as a function of age variability, all rabbit samples were combined for subsequent determination of statistical differences in type II collagen and aggrecan gene expression levels. Pooled data from rabbit samples of both ages showed again no statistically significant differences in equivalence in gene levels between the three anatomic regions investigated in these animals.

The result that the expression levels were not statistically significantly different in equivalence in these several (*n* = 13) individual samples of two distinct ages and growth was unexpected. In this context, however, it should be noted that the age of rabbit and human bones may be correlated by considering the time of closure of their respective physes. Published literature notes that 1 rabbit week = ~0.59 human years (Kaweblum et al., 1994; Kilborn et al., 2002), so 15- and 19-week-old rabbits equate to humans 8.9 and 11.2 years of age, respectively, a somewhat tight, approximately 2-year age range. The 15- and 19-week-old rabbit bones are not so different in age, then, and the result may not be so surprising that gene expression equivalence was found between the three anatomic sites examined for the study specimens despite combining all thirteen individual bones for genomic analyses.

From this observation, in which thirteen different rabbits from two distinct but closely aged groups each contributed three different physes that yielded equivalence in gene expression levels, it may be suggested as possible that human physes could be found equivalent in a related context. In other words, the physis from one patient could serve as a control for comparison with the physis from a patient closely age-matched but with diseased or abnormal bone. Such a comparison has been reported, for example, in the gene expression levels measured between human SCFE (diseased/abnormal bone) and human bone obtained from an epiphysiodesis procedure (control) (Scharschmidt et al., 2009).

Histology and IHC staining for proteoglycans and type II collagen, respectively, showed no qualitative differences between anatomic regions comprising the three different physes of the several rabbit bones examined. The many specimens from the animals were grossly similar and revealed no marked morphologic differences in cell or matrix structure or physical character. There was also statistically established equivalence in histomorphometric measures of physeal height/thickness in the three different tissue regions investigated in the rabbits. These data suggest that physeal tissue from different anatomic regions in rabbit are effectively equivalent and can be used as control tissue in the design of experiments where the gross characteristics of the growth plate may be altered as in pathological conditions like SCFE or hypothyroidism.

Gene expression analyses of type II collagen and aggrecan also demonstrated statistical equivalence among the same anatomic regions of the normal rabbits. Type II collagen provides shape and tensile strength to growth plate cartilage and is the primary collagen in this tissue (Maes and Kronenberg, 2012; Ballock and O'Keefe, 2003). Reports by Agamanolis et al. (Agamanolis et al., 1985a; Agamanolis et al., 1985b) and Ippolito et al. (Ippolito et al., 1981) documented abnormal collagen fibrils in SCFE and suggested such fibrils were likely the source of the weakened physes in this condition. Collagen gene expression levels play a role in fibril structure, organization, and additional features of the protein. It is possible that abnormal type II collagen expression would lead to the observed femoral disorganization in SCFE growth plates or other possible pediatric growth plate disorders. In the normal rabbit bone models studied in this investigation, the results here show healthy growth plates from three different anatomic sites have statistically equivalent expression of type II collagen and aggrecan and therefore one site in a bone could be used as a control to investigate type II collagen and aggrecan abnormalities from another growth plate location.

Aggrecan is a large proteoglycan molecule that is the most abundant of the cartilaginous proteoglycans and provides osmotic properties to the cartilage matrix that support the resistance of compressive loads (Maes and Kronenberg, 2012; Ballock and O'Keefe, 2003; Hunziker, 1994). Little et al. (Little et al., 2005) have demonstrated a link between aggrecan abnormalities and growth plate defects and Scharschmidt et al. (Scharschmidt et al., 2009) showed a definitive decrease in both aggrecan and type II collagen gene expression in SCFE proximal femur growth plates. Further, the association between aggrecan and type II collagen gene expression and physeal defects has been reported by Tank et al. (Tank et al., 2013) and Bries et al. (Bries et al., 2012) using miniature swine and rabbit models, respectively.

The technology of LCM has refined expression data obtained from bulk samples and cell populations by allowing the isolation of individual cells or small cell clusters from sections of tissue (Bonner et al., 1997; Emmert-Buck et al., 1996; Simone et al., 1998). The remarkable improvement in cell selectivity and sampling by LCM is particularly helpful in innumerable studies, including those in the growth plate, as it now yields expression results in molecular biological detail at the single cell level (Jacquet et al., 2005; Kim et al., 2003; Shao et al., 2006). LCM coupled with microarray analysis is a powerful tool to gain understanding in growth plate physiology. For example, LCM has been utilized to isolate cell populations in hypertrophic and proliferative zones of normal rat and mouse growth plates and to characterize them for gene expression differences in these zones by microarray analysis (Wang et al., 2004; Lui et al., 2018). It should be noted that gene expression analyses of the growth plate are especially limited in humans where tissue is simply unavailable or only one human tissue sample may be obtained from a surgical procedure and utilized to compare to another provided from a clinical pathology. As an example, with respect to physeal investigations, a previous LCM study of human SCFE by Scharschmidt et al. (Scharschmidt et al., 2009) examined normal/control tissues obtained from proximal tibia epiphysiodesis surgery to verify consistency of two different growth plate locations, an approach that is also supportive of the data presented here. Further, microarray data from rare SCFE samples when compared to these human control tissues show great promise toward gaining further insight into the condition (Johnson et al., 2016). This present study in rabbit compared three separate growth plate sites and established a statistical equivalence in type II collagen and aggrecan gene expression by a limited LCM growth plate isolation and a larger sampling of ground bones.

There are notable limitations to this study. To begin, this animal model for skeletal research is considered more suitable than rodent models because of growth plate closure in rabbits, but its results may not be easily extrapolated to human tissues (Allen, 2017). Second, the application of histology and IHC analysis was restricted to qualitative assessments of tissue staining for proteoglycans and type II collagen, respectively. While these are the two principal structural components in physes, other molecules could be documented as well. Another apparent limitation to the present investigation was that the rabbit groups were of two different ages. Age variability, however, did not affect gene expression results as there were no statistically significant differences when the two ages of the animals were compared to each other. Pooled data from rabbit samples of both ages showed again no statistically significant differences in gene levels between the three anatomic regions investigated. This study was limited to assessment of only two genes from among many others that could be examined. As major extracellular matrix components, the expression of type II collagen and aggrecan, nonetheless, is well recognized to play a major role in the structure and function of the growth plate. Finally, equivalence is a difficult statistical parameter to evaluate because most studies are designed to analyze statistical differences between a normal/control and a diseased or otherwise altered specimen. Initially, this report determined that there were no detectable structural or statistically significant gene expression differences (in type II collagen and aggrecan) between the anatomic regions within the species with sufficient power for detecting a two-fold statistical difference in gene levels.

The long bones obtained here from the same animal were normal, and interchanging one available physis to be used as an equivalent control required a more stringent statistical evaluation. The setting of equivalency parameters in this case required determination. In this study, equivalency values for morphometry and gene expression were based on literature results of statistically significant differences (Tank et al., 2013) since, to the knowledge of the authors, the present work is the first to try to establish limits for statistically significant equivalence between long bone growth plates. Another recent study has shown statistical differences in growth plate height less than the parameter set here for a 1.8-fold change, but that report analyzed recovery from a growth plate insult and may not have reflected values for equivalency (Nakase et al., 2015). Certainly, a better understanding of the establishment of parameter value limits such as those for growth plate height (thickness) would benefit from additional analyses of equivalency from different growth plates in different species.

The analytical results detailed here are consistent in demonstrating that physes from three distinct anatomic sites in rabbits yield no qualitative differences in histology or immunohistochemistry and demonstrate statistical equivalence in long bone growth plate height and in gene expression levels of type II collagen and aggrecan. These data suggest that the three regions are equivalent and support the concept of utilizing any of them as control tissue for comparing and assessing bone pathologies within a single species. The data appear also to support an assumption made by previous authors (Scharschmidt et al., 2009) that healthy growth plates from different regions, particularly those of long bones, can be used for comparative growth plate studies. Further studies with other approaches to histology, immunohistochemistry and microarray analysis and comparison of physeal structure and greater numbers of genes than the two investigated here would be helpful to confirm the principal conclusions that normal growth plates in a single species are equivalent regardless of their anatomic location.

It should be noted that the initial concept of this study was not to assess and possibly establish statistical equivalence among various physes in rabbits, and indeed the work is not to be construed as focused on either rabbits or any other animal model. Instead, the rationale for this investigation was to initiate research into understanding potential equivalence between readily available tissues in animals with the hope that an extrapolation of data could be made to humans. Since normal or control tissues in humans are very difficult to obtain, the idea was thereby explored that tissue from a surgical procedure from one patient could be utilized as a control for comparison with another patient diagnosed with an abnormality in a related tissue. As mentioned above, such a correlation has been made in at least one instance with bone harvested from two different patients, in which a SCFE biopsy was compared to bone retrieved from an epiphysiodesis (Scharschmidt et al., 2009).

In summary, this work represents the first analysis of physeal structure and gene expression to document equivalence of three different anatomic regions (proximal and distal femur and proximal tibia) within a species (rabbit). The study uniquely applies morphological, histological, immunohistochemical, gene expression, and statistical analyses as means to explore such equivalence. Extrapolation of the validation of equivalence in rabbit physes may be particularly appropriate and important in human orthopedic studies where comparable physeal normal/control tissue is not easily obtained.

## Transparency Document

Transparency document Image 1
